# Factors associated with participation in an ongoing national catch-up campaign against rubella: a cross-sectional internet survey among 1680 adult men in Japan

**DOI:** 10.1186/s12889-021-10340-8

**Published:** 2021-02-04

**Authors:** Ai Hori, Shiho Yoshii, Yukari Isaka, Koji Wada

**Affiliations:** 1 Department of Global Public Health, Faculty of Medicine, University of Tsukuba, 1-1-1 Tennodai, Tsukuba City, Ibaraki 305-8577 Japan; 2 Department of Public Health, Faculty of Medicine, Graduate School of Medicine and Public Health, International University of Health and Welfare, 26-1 Akasaka-4chome Minato-ku, Tokyo, 107-8402 Japan

**Keywords:** Rubella, Antibody test, Adult, Immunization, Japan

## Abstract

**Background:**

Since 2019, aiming to eliminate periodic rubella outbreaks, the Japanese government has provided a rubella immunization program targeting men born in fiscal years 1972 to 1978, who lacked the opportunity to be vaccinated against rubella in childhood. This study aimed to explore the factors associated with participation in the rubella vaccination program among the first-year target population in 2019.

**Methods:**

A total of 11,754 adult men in Japan born in fiscal years 1972 to1978 living in seven rubella epidemic areas (Tokyo, Chiba, Kanagawa, Saitama, Aichi, Osaka, and Fukuoka) were selected from a list of a survey agency and invited to complete an Internet questionnaire in March 2020. Recruitment ended when the participants reached 1680 individuals. Multivariable log binomial regression analyses were performed to explore the association between awareness of rubella prevention and rubella antibody testing in fiscal year 2019, adjusting for social characteristics.

**Results:**

Of the 1680 men aged 41–47 years who completed the survey, approximately half (51.3%) said that they had received a voucher for the rubella antibody testing and vaccination program. One-quarter (25.9%) of the respondents had used the voucher for rubella antibody testing in 2019, and 6.0% had used the voucher for rubella vaccination in fiscal year 2019. Respondents who understood the government recommendation for rubella antibody testing and vaccination for men of their generation (odds ratio [OR]: 5.50; 95% confidence interval [CI]: 4.01–7.53), those with acquaintances who had undergone rubella testing (OR: 1.39; 95% CI: 1.22–1.59), and those who knew that about their lack of opportunity for rubella vaccination (OR: 1.33; 95% CI: 1.11–1.60) tended to undergo rubella antibody testing. Receiving the most recent seasonal influenza vaccination (OR: 1.26; 95% CI: 1.10–1.43) and being able to confirm a rubella vaccination history (OR: 1.28; 95% CI: 1.13–1.46) were also associated with rubella antibody testing.

**Conclusions:**

The ongoing Japanese test-and-vaccinate rubella program has yet to achieve its participation rate goal for 2019. Further dissemination of the government recommendation to the population is necessary, along with improvements in the accessibility of the rubella vaccination program.

**Supplementary Information:**

The online version contains supplementary material available at 10.1186/s12889-021-10340-8.

## Background

Japan periodically faces rubella outbreaks among adult men who did not have the opportunity to be immunized against rubella in childhood [1]. In 2013, a rubella outbreak in Japan resulted in 14,344 reported rubella cases and 45 newborns with congenital rubella syndrome [2]. At the time, the Japanese government set the goal of eliminating rubella by 2020 [3] and recommended voluntary rubella antibody testing or vaccination for women intending to conceive, as well as for their family members [4]. However, another rubella outbreak occurred in 2018–2019, mainly among adult men who contracted the virus in the workplace [5]. Erratic poor rubella immunity among adult men in Japan is a consequence of the country’s policy. The initial Japanese rubella-containing vaccination policy was only introduced for junior high school-aged girls born in fiscal years 1962 to 1978 for the purpose of prevention of congenital rubella syndrome. Boys born in those same years did not have the opportunity to be vaccinated [5].

Strengthening the immunization system is critical for eliminating rubella, but many countries continue to struggle with improving vaccination uptake. Rubella elimination can be achieved through a combination of routine childhood immunization and the vaccination of people in older age groups who are susceptible to the virus (“speed-up” campaigns) [6]. Although all six World Health Organization regions had the goal of eliminating rubella by 2020, rubella elimination has thus far only been achieved in the Americas [7].

Recently, the Japanese government decided to implement a unique rubella immunization program in response to the rubella outbreak in 2018–2019, targeting susceptible men born from fiscal years 1962 to 1978, who did not have the opportunity to be vaccinated against rubella as children [8, 9]. Under the new program, free vaccination is provided for participants with negative rubella antibody test results (i.e., < 8 IU/ml using the hemagglutination inhibition method) [5]. Beginning in February 2019, in the first year of the 3-year speed-up program, vouchers for free antibody testing were mailed to all men in Japan born from fiscal years 1972 to 1978. This initial group comprised half of the total target population of the campaign, with the other half (i.e., men born from fiscal years 1962 to 1971) to receive free antibody testing vouchers in fiscal year 2020 or after, according to the Japanese government’s strategy [9]. A simulation study [10] concluded that the cost of serological testing made it inferior to a random vaccination policy, but a test-and-vaccinate policy may reduce the number of rubella cases if the target population participates in the program. However, the Japanese government’s speed-up campaign against rubella has faced the problem of low participation rates. Despite the municipal office having already sent vouchers by postal mail directly to the target population’s residences, only 21.2% (approximately 1.34 million of 6.46 million) men in the target population for the first year of the program had been tested for rubella antibodies by the end of fiscal year 2019 [11].

To accelerate rubella vaccination coverage among the susceptible population in Japan, it is necessary to identify the barriers to this population’s participation in the vaccination program. Therefore, our study aimed to explore the factors associated with participation in the rubella vaccination program among the first-year target population in 2019: men born from fiscal years 1972 to 1978.

## Methods

### Data collection

A total of 1680 adult men were recruited for this study from the registry of a web-based survey company (INTAGE Inc., Tokyo, Japan). In late March 2020, this survey company randomly selected persons from a list of 11,754 individuals (1) aged 41–47 years and (2) who lived in one of seven prefectures in Japan (Tokyo, Chiba, Kanagawa, Saitama, Aichi, Osaka, or Fukuoka) then invited them to participate in the study. We selected these prefectures with large metropolitan areas, because the latest rubella epidemic impelled their local governments to actively promote rubella countermeasures in the target population. Those who agreed to participate were subsequently directed to complete an anonymous online, self-administered questionnaire. Registrants were provided with financial incentives for their participation. Recruitment ended when the number of participants reached approximately 1600 individuals. The sample size was calculated if the margin of error was 0.5, proportion of rubella antibody testing was 0.21, and response rate was 0.15 among the population (*n* = 6.46 million), with 95% confidence intervals.

### Questionnaire

#### Demographic information

The survey questions included basic demographic information such as age, gender, marital status (married or unmarried), highest level of education completed (less than high school, college or vocational school, university or higher, or other/prefer not to answer), household income (< 5, 5–7.99, or ≥ 8 million Japanese yen/year, or do not know/prefer not to answer), number of children (≤ 1, or > 1), and occupation (employee/civil servant as a regular employee, manager, non-regular employee, or self-employed/other). Participants were also asked about their smoking status (never, current, or former smoker). To assess partner’s current desire for pregnancy, each participant was asked the following question: “Does your spouse (partner) currently have a desire to become pregnant?” with the response options of *yes*, *no*, and *do not know*. To determine the respondents’ vaccination behavior, they were asked, “Did you get vaccinated against influenza this season?” with the response options of *yes*, *no*, and *do not know*. We developed the original questionnaire in Japanese for this study. The English translated version of the questionnaire is available (see Additional file 1).

#### Outcomes

To determine the respondents’ rubella testing and vaccination history from February 2019 to March 2020 (i.e., fiscal year 2019), they were asked, “Did you receive a voucher for rubella antibody testing and vaccination from your residential local government?” (response options: *yes*, *no*, *do not know*), “Did you receive an antibody test for rubella using the voucher?” (response options: *yes*, *no*), and “Did you get a rubella vaccination using the voucher?” (response options: *yes*, *no*).

#### Awareness of rubella prevention

To assess the respondents’ understanding of the government recommendation regarding rubella vaccination for men of their generation, we asked, “Are you aware that it is recommended that men born from fiscal years 1962 to 1978 receive a rubella vaccination?” (response options: *yes*, *no*). To determine whether an individual’s rubella vaccination history could be confirmed, we asked, “At present, do you have access to your records such as the Maternal and Child Health Handbook (not including your parents’ recollection) to confirm whether or not you have previously been vaccinated against rubella?” (response options: *yes*, *no*). Each participant was also asked, “Do you have acquaintances who received a rubella antibody test from February 2019 to March 2020?” to determine whether the respondents’ acquaintances were tested for rubella antibodies in fiscal year 2019. The responses were categorized as none *no* or *yes* in the analysis.

The participants were also asked the following questions to measure their perceptions of the risks associated with a rubella outbreak: “Are you aware that men in your generation, born from fiscal years 1962 to 1978, had no opportunity to be vaccinated against rubella?” (response options: *yes*, *no*) and “Are you aware that babies carried by mothers who are infected with rubella may develop a serious condition called congenital rubella syndrome?” (response options: *yes*, *no*).

### Statistical analysis

The proportions of the participants who received a rubella voucher, underwent rubella antibody testing, and were vaccinated against rubella in fiscal year 2019 and the 95% confidence intervals (CIs) were calculated by the assessed participant characteristics. Log binomial regression analyses were performed to explore the associations between participants’ awareness of the rubella prevention program and their history of rubella antibody testing in fiscal year 2019, adjusting for social background characteristics. All demographic variables (except for gender and age) and variables shown to be statistically significant (*P* < 0.05) in the univariate analyses were included in the primary model, and the variables that remained significant were then selected for the final model. Using a similar model, we also assessed receipt of a voucher for the rubella antibody testing and vaccination program as the dependent variable. All analyses were done with Stata/MP, Version 16.1 (College Station, TX, USA). Statistical tests were two-sided and regarded as statistically significant at *P* < 0.05.

### Ethics statement

This study was approved by the ethical committee of the International University of Health and Welfare (19-Im-013).

## Results

Table 1 shows the characteristics of the study participants. Among men born in fiscal years 1972 to 1978 and who live in a metropolitan area in Japan, 33.0% were current smoker, 77.6% were regular employee or managers, 55.8% were married, and 40.5% were living with children, showing equal to the national survey.

**Table 1:** Characteristics of the study participants (*n* = 1680)^a^

	n (%)
Smoking status	
Never smoked	670 (39.9)
Current smoker	554 (33.0)
Former smoker	456 (27.1)
Education completed	
Less than high school	490 (29.1)
College or vocational school	299 (17.8)
University or higher	847 (50.4)
Other or prefer not to answer	44 (2.6)
Work status	
Regular employee	1017 (60.5)
Manager	287 (17.1)
Non-regular employee	167 (9.9)
Self-employed or other	209 (12.4)
Household income per year	
< 47,000 US dollars^b^	241 (14.4)
47,000–75,000 US dollars	488 (29.1)
> 75,000 US dollar	533 (31.7)
Do not know or prefer not to answer	418 (24.9)
Current marital status	
Married	938 (55.8)
Partner’s current desire for pregnancy	
Yes	205 (12.2)
Living with children	
Yes	680 (40.5)
Current influenza vaccination	
Yes	572 (34.0)
No	957 (57.0)
Do not know	151 (9.0)

^a^Men aged 41–47 years in Japan in 2019

^b^1 US dollar = 106 Japanese Yen (2020)

Of a total of 1680 men aged 41 to 47 years, approximately half (51.3%) said that they had received a voucher for the rubella antibody testing and vaccination program (Table 2). One-quarter (25.9%) of all study participants had used this voucher to undergo rubella antibody testing in fiscal year 2019, and 6.0% of the total study sample were vaccinated against rubella in fiscal year 2019. The Japanese government’s recommendation that men born from fiscal years 1962 to 1978 receive a rubella vaccination was understood by 962 (57.3%) of the respondents.

**Table 2:** Awareness of rubella and participation in the rubella vaccination program^a^

	n (%)
Knowledge about rubella	
Understood the government recommendation about rubella vaccination for men of their generation	962 (57.3)
Knew that men born from fiscal years 1962 to 1978 had no opportunity for rubella vaccination	831 (49.5)
Had acquaintances who received the rubella antibody test or vaccination	138 (8.2)
Able to confirm a rubella vaccination history by the Maternal and Child Health Handbook	360 (21.4)
Aware of congenital rubella syndrome	913 (54.4)
Participation in the rubella vaccination program	
Received a voucher for rubella antibody testing and vaccination from the government in fiscal year 2019	862 (51.3)
Underwent rubella antibody testing in fiscal year 2019 using the voucher	435 (25.9)
Vaccinated against rubella in fiscal 2019 using the voucher	101 (6.0)

^a^Among men aged 41–47 years in Japan in 2019 (*n* = 1680)

The percentages (95% CIs) receiving a voucher, undergoing rubella antibody testing, and receiving the vaccination varied by subgroup (Table 3). Those who did not understand the government’s rubella vaccination recommendation had the lowest percentages reporting that they had received a voucher (17.0% [14.3–19.9%]), underdone rubella antibody testing (4.7% [3.3–6.6%]), and received the vaccination (0.42% [0.09–1.2%]). The highest levels of awareness of receiving a voucher (88.4% [81.9–93.2%]) and undergoing antibody testing (63.0% [54.4–71.1%]) were observed among respondents who had acquaintances who had undergone rubella antibody testing.

**Table 3:** Voucher receipt, rubella antibody testing, and vaccination in fiscal year 2019 by individual characteristics^a^

Variable	n	Received a voucher % (95% CI) (*n* = 862)	Rubella antibody testing % (95% CI) (*n* = 435)	Rubella vaccination % (95% CI) (*n* = 101)
Total	1680	51.3 (48.9–53.7)	25.9 (23.8–28.1)	6.0 (4.9–7.3)
Understood the government recommendation				
Yes	962	76.9 (74.1–79.6)	41.7 (38.5–44.9)	10.2 (8.3–12.3)
No	718	17.0 (14.3–19.9)	4.7 (3.3–6.6)	0.42 (0.09–1.2)
Aware of their lack of opportunity for rubella vaccination				
Yes	831	70.6 (67.4–73.7)	40.2 (36.8–43.6)	10.0 (8.0–12.2)
No	849	32.4 (29.3–35.7)	11.9 (9.8–14.3)	2.1 (1.3–3.3)
Had acquaintances who received the rubella antibody test				
Yes	138	88.4 (81.9–93.2)	63.0 (54.4–71.1)	10.9 (6.2–17.3)
No	1542	48.0 (45.5–50.5)	22.6 (20.5–24.7)	5.6 (4.5–6.8)
Able to confirm a rubella vaccination history				
Yes	360	70.3 (65.3–75.0)	41.9 (36.8–47.2)	11.9 (8.8–15.8)
No	1320	46.1 (43.4–48.9)	21.5 (19.3–23.8)	4.4 (3.4–5.6)
Aware of congenital rubella syndrome				
Yes	913	64.4 (61.2–67.5)	35.3 (32.2–38.5)	8.0 (6.3–9.9)
No	767	35.7 (32.3–39.2)	14.7 (12.3–17.4)	3.7 (2.4–5.2)
Smoking status				
Never	670	56.7 (52.9–60.5)	29.3 (25.8–32.9)	7.3 (5.5–9.6)
Current	554	43.9 (39.7–48.1)	20.0 (16.8–23.6)	3.1 (1.8–4.9)
Former	456	52.4 (47.7–57.1)	28.1 (24.0–32.4)	7.7 (5.4–10.5)
Partner’s current desire for pregnancy				
Yes	205	59.0 (52.0–65.8)	34.6 (28.1–41.6)	8.3 (4.9–12.9)
No	1475	50.2 (47.7–52.8)	24.7 (22.5–27.0)	5.7 (4.6–7.0)
Current influenza vaccination				
Yes	572	64.7 (60.6–68.6)	39.7 (35.7–43.8)	10.5 (8.1–13.3)
No	957	47.2 (44.0–50.5)	19.3 (16.9–22.0)	3.8 (2.6–5.2)
Do not know	151	26.5 (19.6–34.3)	15.2 (9.9–22.0)	3.3 (1.1–7.6)

*CI* Confidence interval

^a^Among men aged 41–47 years in Japan (*n* = 1680)

Table 4 presents the results of the binomial logistic regression analysis for two dependent variables: undergoing rubella antibody testing and receiving a voucher. Respondents who understood the government recommendation had greater odds of undergoing rubella antibody testing than did those who did not understand this recommendation (odds ratio [OR]: 5.50; 95% confidence interval [CI]: 4.01–7.53). Respondents who had acquaintances who had received a rubella test (OR: 1.39; 95% CI: 1.22–1.59) and those who knew that they themselves had not had the opportunity to be vaccinated against rubella (OR: 1.33; 95% CI: 1.11–1.60) had relatively high odds of undergoing rubella antibody testing. Turning to the analysis of receiving a voucher as the dependent variable, similar tendencies were observed for understanding the government recommendation (OR: 4.00; 95% CI: 3.36–4.77), having acquaintances who had received a rubella test (OR: 1.19; 95% CI: 1.11–1.28), and knowing that they had not had the opportunity to be vaccinated against rubella (OR: 1.12; 95% CI:1.02–1.22). Having received the most recent seasonal influenza vaccination (OR: 1.26; 95% CI: 1.10–1.43) and being able to confirm a rubella vaccination history (OR: 1.28; 95% CI: 1.13–1.46) were also significantly associated with undergoing rubella antibody testing. Current smokers (OR: 0.89; 95% CI: 0.82–0.97) had significantly lower odds of receiving a voucher than did those who did not currently smoke. Because awareness of congenital rubella syndrome was not significantly associated with either dependent variable in the multivariable analyses, this variable was omitted from the final models.

**Table 4:** Log binomial regression analysis predicting voucher receipt and rubella antibody testing in fiscal year 2019^a^

	Received a voucher				Tested rubella antibody			
	OR	95% CI	AOR	95% CI	OR	95% CI	AOR	95% CI
Understood the government recommendation	4.53	(3.84–5.34)	**4.00**	**(3.36–4.77)**	7.87	(5.87–10.56)	**5.50**	**(4.01–7.53)**
Aware of lack of opportunity for rubella vaccination	2.18	(1.96–2.43)	**1.12**	**(1.02–1.22)**	3.23	(2.70–3.87)	**1.33**	**(1.11–1.60)**
Had acquaintances who received the rubella antibody test	1.84	(1.70–1.96)	**1.19**	**(1.11–1.28)**	2.77	(2.43–3.16)	**1.39**	**(1.22–1.59)**
Able to confirm a rubella vaccination history	1.52	(1.39–1.66)	1.00	(0.94–1.07)	2.27	(1.98–2.60)	**1.28**	**(1.13–1.46)**
Smoking status								
Never	ref		ref		ref		ref	
Current	0.77	(0.69–0.87)	**0.89**	**(0.82–0.97)**	0.69	(0.57–0.83)	0.88	(0.75–1.02)
Former	0.92	(0.83–1.03)	0.95	(0.88–1.02)	0.98	(0.83–1.16)	0.97	(0.86–1.09)
Partner’s current desire for pregnancy								
No	ref		ref		ref		ref	
Yes	1.17	(1.04–1.33)	0.97	(0.89–1.06)	1.49	(1.24–1.78)	0.98	(0.87–1.11)
Current influenza vaccination								
No or do not know	ref		ref		ref		ref	
Yes	1.46	(1.33–1.59)	1.06	(0.99–1.13)	2.05	(1.78–2.36)	**1.26**	**(1.10–1.43)**

Both models were adjusted for education, marital status, and all variables listed in the table

*OR* Odds ratio, *AOR* Adjusted odds ratio, *CI* Confidence interval, *ref* Reference category

^a^Among men aged 41–47 years in Japan (*n* = 1680)

Table 5 shows the results regarding rubella antibody testing among the survey participants. Of participants who were recommended to undergo rubella vaccination based on the antibody testing results (*n* = 104), 90% had already received their vaccination at the time of the survey.

**Table 5:** Rubella antibody testing result among study participants (*n* = 435)

	n (%)
Place of testing rubella antibody level	
Clinic or hospital	418 (96)
Health checkup	12 (3)
Others	5 (1)
Result of rubella antibody testing	
Not recommended vaccination according to the antibody level	307 (71)
Recommended rubella vaccination	104 (24)
Not check the test result yet	22 (5)
Do not remember or cannot understand the result	2 (1)
Complete vaccination among whom recommended rubella vaccination (*n* = 104)	
Vaccinated by using the coupon	94 (90)
Having practical plan for vaccination	4 (4)
No practical plan for vaccination	6 (6)

## Discussion

Approximately one-quarter of the men born from fiscal years 1972 to 1978 responding to the survey had participated in the Japanese government’s rubella catch-up program from February 2019 to March 2020. A national-level report for the same period [11] shows a 21.2% national participation rate was achieved. That is less than half the 51% goal for fiscal year 2019. Japan’s national goal for the catch-up program is to increase the percentage of men born in fiscal years 1962 to 1978 and who have rubella antibodies from the baseline 80% (in 2018) to 90% by the end of March 2022 [9]. Policymakers are primarily concerned with the worldwide alert concerning rubella outbreaks in Japan [12], especially in the run-up to the (postponed and rescheduled) Tokyo 2020 Olympic/Paralympic Games, as the number of overseas visitors to Japan is expected to increase [9]. The government, recognizing the late start of the catch-up program, already began to promote boosting the speed-up strategy by including rubella antibody testing in annual health check-ups provided in the workplace in early 2020 [13].

The present study suggests that men’s understanding of the necessity of rubella vaccination for their own health promotes their participation in the rubella prevention program. However, such knowledge is not yet widespread among men born from fiscal years 1972 to 1978 (Table 2). Understanding the government recommendation for rubella vaccination and being aware of their own lack of opportunity to be vaccinated against rubella were found to be substantial factors associated with both reporting that they had received a voucher and undergoing testing for rubella antibodies, adjusting for other variables in the model (Table 4). Being able to confirm a rubella vaccination history was also significantly associated with antibody testing. These results are consistent with our previous report of the results of a 2014 survey [14]. In the present study, we also found that awareness of congenital rubella syndrome was not significantly associated with rubella antibody testing in the multivariate analyses. This finding suggests that the target population took preventive action for themselves, rather than to benefit others. Furthermore, having acquaintances who had been tested for rubella antibodies promoted positive attitudes toward rubella vaccination. Distributing information via social media or in workplaces about the potential benefit of rubella antibody testing for adult men may improve their participation rate. Previous reports have also suggested that social relationships [15] and social norms in the workplace [16] are associated with getting vaccinated among adults. Positive attitudes toward rubella vaccination may be amplified among people who share information about the need to be vaccinated.

Although the participation rate of Japan’s new rubella vaccination program remains low, the public recognition of the new rubella vaccination policy has slightly changed. We found evidence that knowledge is increasing regarding the need for adult men to be vaccinated against rubella, rather than only women and babies needing vaccination. The present study found that partner’s current desire for pregnancy did not affect adult men’s participation in the rubella catch-up program. This finding is in contrast to our previous study conducted in 2014, which found that partner’s current desire for pregnancy was a major factor associated with adult men’s voluntary rubella antibody testing and vaccination [14]. This difference in results is reasonable because, at the time of the first survey, during the 2013–2014 rubella outbreak, the government recommendation for voluntary rubella vaccination targeted only women and men with a desire to conceive who could not confirm a rubella vaccination history [4].

The present study revealed two steps that may present obstacles for the rubella catch-up program; 1) receipt of the voucher and 2) taking action using the voucher. The most straightforward strategy to increase the vaccination program participation rate is providing reminders to the target population by telephone, letter, or text message, as a Cochrane meta-analysis has suggested [17]. The reminders local governments sent in 2020 may help in raising awareness about the mailed vouchers. However, the proportion reporting receipt of a voucher was relatively low among men who were current smokers (Table 4). Socioeconomic deprivation is known to be associated with lower levels of knowledge about vaccinations [18]. and vaccine uptake [19] among adults. Current smoking is a factor known to be associated with lower vaccine uptake [19], as is socioeconomic deprivation. Further effort is necessary to encourage participation among hard-to-reach groups. Some European countries have made efforts to provide services that meet participants’ needs and to ensure that most individuals have easy access to vaccination [20]. Previous successful practices suggest that social marketing programs [21] and community-based approaches [22, 23] based on a deep understanding of the target population may be beneficial in achieving the goal of the vaccination program.

Improving access to vaccines is essential for removing barriers and increasing vaccination coverage [24]. Among the men targeted by the rubella catch-up program who participated in our survey, those who did not undergo rubella antibody testing also did not tend to receive the seasonal influenza vaccine (Table 4). The most common reason for not being vaccinated against influenza among employed men in Japan has been reported to be having insufficient time to go to a medical facility [25]. As mentioned above, additional promotion of antibody testing in the workplace health check-up setting [13] has already begun in Japan. Providing rubella antibody testing and blood testing as part of the workplace health check-up, which reaches 80% of the working population in Japan annually, for those aged 40 years or older might improve accessibility, as a previous report has suggested [26].

Our study had a few limitations. First, as an Internet survey population sampled from urban areas, our sample cannot be considered representative of the general Japanese population; thus, the results should be interpreted carefully. The present survey shows a higher participation rate (25.9%) in the rubella catch-up program, compared with a national-level report for the same period (21.2%) [11]. This difference in findings indicates that our survey population may have had more interest in rubella prevention than is the case in the general population. The difference may have caused an overestimation of the rubella antibody testing or vaccination rate, but it would not affect our primary objective of identifying factors associated with program participation. Second, because we used a cross-sectional study design, we were unable to confirm that the findings indicate causal relationships. Despite these limitations, these up-to-date survey results are clearly important to further accelerate the rubella catch-up program in Japan.

## Conclusions

The ongoing Japanese test-and-vaccinate rubella program has yet to achieve half its participation rate goal for 2019, the program’s first fiscal year. Further dissemination of the government recommendation to the population is necessary, along with improvements in the accessibility of the rubella vaccination program, including through incorporating the program in the workplace health check-up setting.

## Supplementary Information

**Additional file 1.** The research questionnaire translated in English (Original version is in Japanese). The original questionnaire in Japanese was developed for this study.

## Data Availability

The datasets used and/or analyzed during the current study are available from the corresponding author on reasonable request.

## Abbreviations

OR: Odds ratio
CI: Confidence interval
