# Supplementary material for: Factors associated with participation in an ongoing national catch-up campaign against rubella: a cross-sectional internet survey among 1680 adult men in Japan
Source: BMC Public Health. 2021 Feb 4;21:292. doi: 10.1186/s12889-021-10340-8 (PMC7863504; doi:10.1186/s12889-021-10340-8)
Supplement: Supplementary file 1 — Additional file 1. The research questionnaire translated in English (Original version is in Japanese). The original questionnaire in Japanese was developed for this study. [file 12889_2021_10340_MOESM1_ESM.docx]

Additional file 1

The research questionnaire translated in English（Original version is in Japanese）

- The following items explain the correct content about "rubella". Do you know the contents?　 (Answer: Yes or No)
  - Are you aware that men in your generation, born from fiscal years 1962 to 1978, had no opportunity to be vaccinated against rubella?
  - Are you aware that babies carried by mothers who are infected with rubella may develop a serious condition called congenital rubella syndrome*? *Congenital rubella syndrome: Innate disorders such as difficulty hearing, heart malformations, difficulty seeing, and delays in mental and physical development.
  - Are you aware that it is recommended that men born from fiscal years 1962 to 1978 receive a rubella vaccination?
  - In the vaccination of rubella that started in February 2019, a blood test (antibody test) is first performed free of charge, and those who are found to have insufficient immunity can be vaccinated free of charge.
- Did you receive a voucher for rubella antibody testing and vaccination from your residential local government?　 (Answer: Yes, No or Do not know)
- At present, do you have access to your records such as the Maternal and Child Health Handbook (not including your parents’ recollection) to confirm whether or not you have previously been vaccinated* against rubella? *Rubella vaccination refers to either the rubella vaccine alone, the measles-rubella combination vaccine (MR vaccine), or the measles-mumps-rubella combination vaccine (MMR vaccine).　 (Answer: Yes or No)
- Did you receive an antibody test for rubella using the voucher after February 1, 2019?　 (Answer: Yes or No)
- To those whom "The antibody titer of rubella was not enough, and vaccination was recommended." Did you get a rubella vaccination using the voucher? (Answer: Yes or No)
- To those who answered, "Immune to rubella was examined by a blood test (antibody test) after February 1, 2019." Did you use the voucher distributed by the local government?　 (Answer: Yes, No or Do not know)
- Do you have acquaintances who received a rubella antibody test from February 2019 to March 2020? (Answer: Yes or No)
- Please answer the following questions about yourself.
  - Does your spouse (partner) currently have a desire to become pregnant? (Answer: Yes, No or Do not know)
  - Did you get vaccinated against influenza this season? (Answer: Yes or No or Do not know)
  - Do you smoke？ (Answer: Currently smoke or Past smoked or Never smoke)
  - Which is your final academic background? (Answer: Junior high school, High school, Junior college, Vocational school, Technical college, University, Graduate school, Others)
  - Which is your current occupation? (Answer: regular employee, civil servant as a regular employee, manager, part-time worker, temporary staff, contract employees, self-employed, student, unemployed, housekeeper, others)
  - Which is your current employment form? (Answer: Regular staff / employees、Parttime worker, temporary staff, contract employees, Self-employed, Unemployed, Others)
  - Which is the tax-included annual income of your household? (Answer: Less than 1, 2, 3, 4, 5, 6, 7, 8, 9, 10, 12, 15, 20 million yen / year, more than 20 million yen / year, Do not know, or Do not want to answer)
  - Which is your birth year (in the fiscal year)? (Answer: 1972, 1973, 1974, 1975, 1976, 1977, 1978)
  - Please tell us your gender（Answer: Men or Women）
  - Please tell us your marital status（Answer: Married, Not married）
  - Please tell us number of the children you living together（Answer: 0, 1, 2, 3, 4 or more）
